# Evaluation and Comparison of Solid Lipid Nanoparticles (SLNs) and Nanostructured Lipid Carriers (NLCs) as Vectors to Develop Hydrochlorothiazide Effective and Safe Pediatric Oral Liquid Formulations

**DOI:** 10.3390/pharmaceutics13040437

**Published:** 2021-03-24

**Authors:** Paola Mura, Francesca Maestrelli, Mario D’Ambrosio, Cristina Luceri, Marzia Cirri

**Affiliations:** 1 Department of Chemistry, University of Florence, via Schiff 6, Sesto Fiorentino, 50019 Florence, Italy; paola.mura@unifi.it (P.M.); francesca.maestrelli@unifi.it (F.M.); 2 Department of Neurofarba, University of Florence, Viale Pieraccini 6, 50139 Florence, Italy; mario.dambrosio@unifi.it (M.D.); cristina.luceri@unifi.it (C.L.)

**Keywords:** solid lipid nanoparticles, nanostructured lipid carriers, hydrochlorothiazide, pediatric therapy, Gelucire, cytotoxicity, cellular uptake

## Abstract

The aim of this study was the optimization of solid lipid nanoparticles (SLN) and nanostructured lipid carriers (NLC) in terms of physicochemical and biopharmaceutical properties, to develop effective and stable aqueous liquid formulations of hydrochlorothiazide, suitable for paediatric therapy, overcoming its low-solubility and poor-stability problems. Based on solubility studies, Precirol^®^ ATO5 and Transcutol^®^ HP were used as solid and liquid lipids, respectively. The effect of different surfactants, also in different combinations and at different amounts, on particle size, homogeneity and surface-charge of nanoparticles was carefully investigated. The best formulations were selected for drug loading, and evaluated also for entrapment efficiency and release behaviour. For both SLN and NLC series, the use of Gelucire^®^ 44/14 as surfactant rather than PluronicF68 or Tween^®^ 80 yielded a marked particle size reduction (95–75 nm compared to around 600–400 nm), and an improvement in entrapment efficiency and drug release rate. NLC showed a better performance than SLN, reaching about 90% entrapped drug (vs. 80%) and more than 90% drug released after 300 min (vs. about 65%). All selected formulations showed good physical stability during 6-month storage at 4 °C, but a higher loss of encapsulated drug was found for SLNs (15%) than for NLCs (<5%). Moreover, all selected formulations revealed the absence of any cytotoxic effect, as assessed by a cell-viability test on Caco-2 cells and are able to pass the intestinal epithelium as suggested by Caco-2 uptake experiments.

## 1. Introduction

Drugs belonging to the cardiovascular group are largely utilized in paediatric therapy, due to their several indications in severe and/or chronic clinical conditions such as heart failure, hypertension, hypovolemic shock or oedema. However, the European Medicines Agency (EMA) pointed out that a limited number of antihypertensive drugs is available in suitable formulation for paediatrics [1]. Among these, hydrochlorothiazide (HCT) is one of the diuretic drugs most widely used in the treatment of paediatric hypertension, being also present in the World Health Organization (WHO) Model List of Essential Medicines for Children [2]. This is due to the fact that diuretic drugs, and particularly HCT, have a long history of safety and therapeutic efficacy, based on clinical experience in hypertensive children, resulting appropriate for paediatric use [3]. Moreover, the HCT single daily administration makes it easier to be accepted by young patients, thus improving their compliance to the pharmacologic therapy [4].

However, HCT has been classified as a Class IV drug in the Biopharmaceutical Classification System (BCS) due to its poor solubility and low membrane permeability [5,6], often resulting in a low and variable oral availability [7]. Additionally, different studies [8,9,10,11] evidenced problems of stability of this drug in aqueous solutions. For all these reasons, no liquid formulations of HCT are currently present on the market. This gives rise to some limitations in its utilization in paediatrics, where the use of oral solid dosage forms is restricted by their rigid dose content and by the difficulty of children to swallow them [12]. On the other hand, the common practice of caregivers to prepare extemporaneous liquid formulations by crushing of tablets or capsules opening, and then suspending the powder in a suitable liquid, can often lead to loss of accuracy of the dosage, issues with stability, and possible errors due to incorrect manipulation [13,14]. Moreover, excipients present in dosage forms designed for adults could be harmful to children, particularly for neonates and infants [15,16]. Therefore, the development of a stable HCT liquid formulation suitable for paediatric use would allow to overcome all the above drawbacks and make its therapeutic use easier, safer and more effective.

Lipid-based nanocarriers emerged as a powerful approach to enhance the stability and oral bioavailability of poorly soluble drugs. Moreover, in virtue of their biocompatibility, biodegradability and absence of toxicity [17,18], they can be considered suitable also for pediatric formulations. Among these carriers, solid lipid nanoparticles (SLNs), formed by a dispersion of solid, biocompatible lipids in an aqueous phase, proved to be a valid alternative to liposomes, providing similar benefits in terms of safety in use, but overcoming some of their main disadvantages, such as the limited stability and poor entrapment efficiency [19,20]. Furthermore, the solid lipid matrix can offer a better protection of the entrapped drug [21]. An oral liquid pediatric formulation of HCT loaded in SLNs has recently been proposed, endowed with satisfactory encapsulation efficiency (near to 60%) and acceptable storage stability (1 month) [22].

Nanostructured lipid carriers (NLCs) represent another interesting type of lipid nanocarrier, planned with the purpose of improving some potential shortcomings presented by SLNs, especially their propensity to throw out the drug from the matrix during the storage, due to the highly ordered status of the crystalline solid lipid [23]. In fact, differently from SLNs, the solid matrix of NLCs is formed by a mixture of biocompatible solid and liquid lipids, thus giving rise to a less-ordered, imperfect structure, which should ensure improved physical stability, higher drug loading ability and lower tendency to prematurely leak the drug during storage [24]. Actually, a NLC-based oral liquid pediatric formulation of HCT recently developed proved to be more effective than the previous SLN drug formulation, both in terms of drug entrapment efficacy (around 90% vs. 60%) and storage stability (3 months vs. 1 month) [25].

Considering these interesting and promising results, it seemed worthy of interest to further investigate and compare the effectiveness of SLNs and NLCs as carriers for the development of suitable oral liquid pediatric formulations of HCT, by particularly focusing the attention on the proper choice of the most effective solid and liquid lipid components and carefully investigating the effect of different surfactants, also in combinations and at different amounts, in order to optimize the nanoparticle properties (in terms of size, homogeneity, entrapment efficiency and storage stability). Cytoxicity and cellular uptake studies were finally performed using Caco-2 cells, in order, respectively, to verify the safe use of the selected formulations, and to assess their ability to enter the intestinal epithelium cells.

## 2. Materials and Methods

### 2.1. Materials

Hydrochlorothiazide (HCT) was a generous gift of Menarini (Florence, Italy). Glyceryl distearate/palmitostearate (Precirol^®^ ATO5), high-purity diethylene glycol monoethyl ether (Transcutol P), highest-purity diethylene glycol monoethyl ether (Transcutol^®^ HP), glyceryl dibehenate (Compritol^®^ 888 ATO), lauroylpolyoxyl-32-glycerides (Gelucire^®^ 44/14), glyceryl monostearate 40/55 (Type I) (Geleol^®^), cetyl palmitate, caprylic/capric triglycerides (Labrafac^®^ Lipophile WL 1349), PEG-8-caprylic/capric glycerides (Labrasol^®^) and caprylocaproyl polyoxyl-8 glycerides (Labrasol ALF), were kindly provided by Gattefossé (Cedex, France). Glyceryl tripalmitate (Tripalmitin) and Rhodamine 6G were from Sigma Aldrich (St. Louis, MO, USA), glyceryl monostearate (Imwitor^®^ 491) and medium chain (C8-C10) triglycerides (Miglyol^®^ 810N) were kindly provided by Cremer Oleo GmbH (Witten, Germany). Caprylic triglycerides (Miglyol^®^ 812) was from Sasol GmbH (Witten, Germany), polyoxyethylen-sorbitan monoleate (Tween^®^ 80) from Merck (Hohenbrunn, Germany) and poloxamer 188 (Pluronic^®^ F68) from BASF (Ludwigshafen, Germany). Purified water was obtained by reverse osmosis (Elix 3 Millipore, Rockville, MD, USA). All other chemicals were of reagent grade and used as received.

### 2.2. Screening of Solid and Liquid Lipids

#### 2.2.1. Solubilizing Power of Solid and Liquid Lipids towards the Drug

HCT solubility in different solid and liquid lipids was assessed, in order to select the most effective ones for SLN and NLC preparation.

The solubility in solid lipids was determined by adding 100 mg of HCT to 5 g of the lipid and then heating the obtained mixture until the lipid melted; the drug solubility in the melt lipid was visually estimated by establishing the achievement of a clear solution and the absence of drug crystals [26].

The solubility in liquid lipids was instead determined by adding 100 mg of HCT to 5 mL of liquid lipid and allowing to reach equilibrium (24 h) at 65 °C (to mimic the temperature conditions used during NLC preparation); the drug solubility was then evaluated by visual checking of dissolution of HCT crystals and obtainment of a perfectly transparent, homogeneous system [27].

#### 2.2.2. Compatibility Studies by Differential Scanning Calorimetry (DSC)

Compatibility studies were performed by Differential Scanning Calorimetry (DSC) with a Mettler TA4000 (Star^e^ Software) apparatus (Mettler Toledo, Switzerland) equipped with a DSC 25 cell. Samples were accurately weighed (Mettler M3 microbalance) and scanned in pierced Al pans under static air (10 °C/min, 30–300 °C). Indium was used as a standard (99.98% purity; T_fus_: 156.61 °C; ΔH_fus_: 28.71 J/g) for temperature and heat flow calibration. Pure drug and components of SLN or NLC and their different combinations at the same ratio used in the final formulation were analysed.

### 2.3. Preparation of Lipid Nanoparticles

SLNs and NLCs were prepared using the Hot High-Shear Homogenization (HSH) technique followed by ultrasonication as described by Cirri et al., 2018 [25]. Briefly, in the case of SLNs, empty nanoparticles were prepared by heating at 65 °C, up to melting the selected solid lipid (5 g). In the meanwhile, the aqueous phase, containing the surfactant (Gelucire^®^ 44/14, Tween^®^ 80, Pluronic^®^ F68, at different %, separately or in mixtures) was heated at the same temperature and then 10 mL were dispersed in the fused lipid by 5 or 10 min homogenization at 10,000 rpm (high shear homogenizer Silverson L5M, Chesham, UK). The pre-emulsion was then subjected to 3 min sonication (amplitude 50%, power 100 W) (Sonopuls HD 2200 sonicator, Bandelin Electronics, Berlin, Germany). The obtained dispersion was finally cooled down to 4 °C.

The procedure for NLCs production was the same, but a liquid lipid was initially added to the solid one at different percentages (0.5, 1, 3 and 5%) and then heated together.

In the case of nanoparticles loaded with HCT, the drug was in all cases added to the lipid phase, so that to have a final concentration of 0.2% *w*/*v*.

All formulations were stored at 4 °C for further investigations.

### 2.4. Characterization of Lipid Nanoparticles (NPs)

#### 2.4.1. Measurement of Particle Size, Polydispersity Index and ζ (Zeta) Potential

Mean diameter and polydispersity index (PDI) of nanoparticles were determined at 25 °C by dynamic light scattering (DLS) using a Zetasizer Nano ZS (Malvern Instruments Ltd., Malvern, UK). Zeta potential (ZP) of nanoparticles was measured by laser Doppler micro-electrophoresis using a Zetasizer Nano ZS (Malvern Instruments Ltd., Malvern, UK). Before measurements, all samples were diluted appropriately with purified water. All analyses were carried out on three samples for each formulation, and the mean value was calculated for each measured parameter.

#### 2.4.2. Determination of Entrapment Efficiency (EE%)

Drug entrapment efficiency was determined by an indirect method, according to Cirri et al., 2012 [28], after separation of the unincorporated drug from drug-loaded nanoparticles by ultrafiltration-centrifugation (centrifugal filters Amicon Ultra-4 with 100 kDa molecular weight cut-off, Millipore, Germany). Briefly, 500 µL of each HCT-loaded nanoparticle dispersion was put into the upper chamber of the ultrafiltration device, and then centrifuged at 12,000 rpm for 30 min. The concentration of unincorporated drug in the aqueous phase, collected in the outer chamber of the ultrafiltration device, was then spectrometrically assayed at 272 nm (UV–vis 1600 Shimadzu spectrophotometer, Tokyo, Japan). Entrapment efficiency (EE%) of HCT in nanoparticles was calculated according to the following equation:

$$EE\%=\frac{W_{total\;drug}-W_{free\;drug}}{W_{total\;drug}}\times 100$$

where *W_free drug_* is the amount of unincorporated drug in the aqueous phase after ultrafiltration-centrifugation. Any interference from other components was observed.

### 2.5. In Vitro Drug Release

In vitro drug release experiments from SLN and NLC were conducted according to the dialysis bag technique [22]. Dialysis bags of cellulose acetate (12,500 cut-off, Sigma-Aldrich, St. Louis, MO, USA) were soaked 12 h in pH 4.5 gastric buffer and then filled with 1 mL of SLN or NLC dispersion. The bags were then immersed, 2 h in 100 mL of pH 4.5 phosphate buffer mimicking the infant gastric pH (HCT solubility in the medium 0.7 mg/mL) and after 3 h in 100 mL of pH 6.8 phosphate buffer (mimicking the intestinal pH), both at 37 °C, under magnetic stirring at 50 rpm. The amount of released drug in the acceptor compartment was UV assayed at 272 nm at given time intervals. Each withdrawn sample was replaced with an equal volume of fresh medium, and a correction for the cumulative dilution was made. Experiments were carried out in triplicate.

Drug release data were fitted into different kinetic models (zero-order, first-order, Higuchi and Korsmeyer–Peppas), in order to find the best fitting kinetic model. The prevalent mechanism of drug release from SLN and NLC samples was also inferred from the value of the diffusional release exponent (n) of the Korsmeyer–Peppas equation:

F_t_ = k × t^n^

where F_t_ is the drug fraction released at time t, k the release constant, n the diffusional exponent.

The values of *n* indicate the mechanism of drug release: *n* < 0.5: Fickian diffusion; 0.5 < *n* < 0.9: non-Fickian transport (anomalous transport), and *n* > 0.9: type-II transport.

### 2.6. Storage Stability Studies of Selected Lipid Nanoparticles

All the selected drug-loaded SLN and NLC dispersions were stored at 4 ± 1 °C for 6 months and checked every 30 days for mean particle size, PDI and zeta potential (Zetasizer Nano ZS, Malvern Instruments Ltd., Malvern, UK). At the end of the storage period, samples were also checked for drug EE%, to evaluate the entity of drug expulsion phenomena. At least three replicate analyses were done for each sample. Possible crystallization, precipitation, mold formation or gelling phenomena were also checked by visual inspection.

### 2.7. Cytotoxicity and Cellular Uptake Studies

Caco-2, a human colorectal adenocarcinoma cell line obtained from American Type Culture Collection (ATCC, Rockville, MD, USA), was cultured in Dulbecco’s Modified Eagle’s medium (DMEM, Euroclone, Milan, Italy), supplemented with 20% fetal bovine serum (FBS), 1% L-glutamine and 1% Penicillin/Streptomycin (all Carlo Erba reagents, Milan, Italy) at 37 °C in an atmosphere containing 5% CO_2_. HCT was dissolved in dimethyl-sulfoxide (DMSO) and, then, diluted in DMEM in order to obtain the appropriate concentrations to be tested. The final concentration of DMSO was <0.1%.

For viability test, Caco-2 cells were seeded in 96-well plates at a density of 5 × 10^3^ cells/well in 100 µL of medium. After 24 h of incubation at 37 °C in 5% CO_2_, the cells were exposed to the formulations, with or without HCT (1:50, 1:100 or 1:200), for 24 h. Cell viability was assessed by a colorimetric method based on reduction of 3-(4,5-dimethylthiazol-2-yl)-5-(3-carboxymethoxyphenyl)-2-(4-sulfophenyl)-2H-tetrazolium, inner salt (MTS, Promega Corporation, Madison, WI, USA). The optical density of the colored formazan product formed by MTS reduction was measured at 490 nm using a VICTOR^3^ Wallac 1421 Multilabel Plate Reader (Perkin Elmer, Ramsey, NJ, USA). Data were expressed as a percentage of viable cells compared to cells exposed to the solvent alone.

For cellular uptake studies, SLN or NLC dispersions were labeled with rhodamine as fluorescence marker, by adding it to the lipid phase to obtain a final concentration of 10^−5^ M. Caco-2 cells were growth on glass cover-slips placed into 4-well plates for 24 h, then the labeled SLN and NLC were added, after 1:50 dilution, and maintained for 1 h at room temperature. After incubation time, cells were washed four times with pH 7.4 phosphate buffer solution (PBS) and fixed in 4% formaldehyde in 0.1 mol/L PBS for 10 min at room temperature. After fixation, cells were washed four times with PBS and cell nuclei were stained with 40,6-diamidino-2-phenylindole (DAPI) for 10 min at room temperature. The cells were washed once with PBS and the glass cover-slips were glued to glass microscope slides and observed by the Olympus BX63 microscope equipped with a Metal Halide Lamp (Prior Scientific Instruments Ltd., Cambridge, United Kingdom) and a digital camera Olympus XM 10 (Olympus, Milan, Italy). Ten photomicrographs were randomly taken for each sample and fluorescence was measured using ImageJ 1.33 image analysis software (freely available at http://rsb.info.nih.gov/ij, accessed on 23 March 2021).

### 2.8. Statistical Analysis

Statistical analysis of data was carried out according to the one-way analysis of variance. Differences between groups were tested by one-way ANOVA with Student-Newman-Keuls comparison post hoc test (GraphPad Prism version 6.0 Software, San Diego, CA, USA). Data were expressed as mean ± SD. A *p* value < 0.05 was considered significant.

## 3. Results and Discussion

### 3.1. Screening of Solid and Liquid Lipids for SLN and NLC Development

A preliminary screening for a proper selection of the solid and liquid lipids to be used for HCT-loaded SLNs and NLCs development was initially performed. It was based on the assessment of the solubility of HCT in the different examined solid and liquid lipids, since this is considered a critical condition for obtaining a good drug loading in the final nanoparticles. The results, in terms of transparency or turbidity of the different solutions after addition of 100 mg of drug to 5 g of melted lipid or 5 mL of liquid lipid, are collected in Table 1. When a transparent solution was obtained, a further 100 mg of drug was added, to achieve the target concentration of 0.2% *w/v* in the final formulation. Among solid lipids, Geleol^®^ and Imwitor^®^ 491 showed a certain solubilizing power towards HCT but only Precirol^®^ ATO5 allowed the total dissolution of this HCT amount, and, among liquid lipids, transparent solutions were obtained only with Transcutol^®^ HP and Transcutol^®^ P.

Based on the results of solubility studies, Precirol^®^ ATO5 was confirmed as the best solid lipid solubilizer for HCT [22] and was then selected for preparation of both SLN and NLC formulations.

On the other hand, Transcutol^®^ HP was selected as the liquid lipid for NLC preparation, due to its higher solvent properties towards the drug than the other tested substances, and it was preferred to Transcutol^®^ P due to its higher purity. In fact, a review about the safety of Transcutol as pharmaceutical excipient proved that the toxicity previously related to high levels of this solvent in nonclinical studies carried out before 1990, is likely to be attributed to the presence of significant amounts of impurities, particularly ethylene glycol; on the contrary, subsequent, extensive literature data showed its long safe use as a vehicle and solvent by multiple routes [29].

Regarding the surfactants, Gelucire^®^ 44/14, considering its amphiphilic nature (HLB 14), was tested as non-ionic surfactant both in alternative to Tween^®^ 80 (HLB 15) and Pluronic^®^ F68 (HLB < 24), used in previous studies [22,25,30,31,32], and also in combination with each of them at different ratios. Gelucire are versatile polymers, consisting in mixtures of glycerides with PEG esters of fatty acids, widely used in drug delivery with different applications, mainly depending on their HLB values [33]. Among the hydrophilic grades of this polymer, Gelucire^®^ 44/14 has been successfully used to improve solubility and dissolution rate of several poorly soluble drugs [34,35,36,37], and it also showed safe absorption enhancer properties [38]. Gelucire^®^ 44/14 is also present in different currently marketed oral lipid-based formulations and is included in the FDA list of inactive ingredients.

Compatibility studies performed by DSC on pure drug and the selected components of SLN and NLC formulations, both separately and in the different combinations used, allowed to rule out possible solid-state interactions and/or incompatibility problems between them, thus confirming their suitability for use (data not shown).

### 3.2. Preparation of Nanoparticles

Preliminary experiments showed that 5 min of high-shear homogenization was enough to obtain particles of nanometric dimensions, while an increase at 10 min of homogenization time did not give rise to a significant reduction in nanoparticle size. Then, 5 min was selected as homogenization time for all nanoparticle formulations.

A series of empty SLN and NLC formulations was then prepared, in order to investigate the effect of different formulation variables on the nanoparticle properties in terms of mean dimensions, homogeneity and Zeta potential.

In particular, in the case of SLN formulations, the effect of replacing 1.5% *w*/*w* of Tween^®^ 80 or Pluronic^®^ F68 with different amounts of Gelucire^®^ 44/14 (from 1.5 up to 7% *w*/*w*), or of their combined use, as well as the effect, by keeping the Gelucire^®^ 44/14 content constant at 3.5% *w*/*w*, of varying the Precirol^®^ ATO5 amount (from 5 up to 20% *w*/*w*), were evaluated.

On the other hand, in the case of NLC formulations, the effect of variations of the liquid lipid Transcutol^®^ HP content (from 0.5 up to 5% *w*/*w*) and of the use of Gelucire^®^ 44/14 as non ionic surfactant instead of, or in combination with Tween^®^ 80 or Pluronic^®^ F68, was investigated.

The composition of the various SLN and NLC formulations is given in Table 2 and Table 3.

### 3.3. Characterization of Nanoparticles Formulations

Particle size, polydispersity index (PDI) and Zeta potential (ZP) were selected as the parameters for nanoparticles characterization, since they represent important characteristics having a strong influence on the stability, release rate and biological performance of the nanoparticulate systems.

In particular, particle size is considered a marker of the system stability and it should maintain a narrow range during storage because an increase in particle size during storage periods indicates agglomeration and hence physical instability [39]. Moreover, it seems that small particle size (particularly below 300 nm) of the formulation should improve the drug bioavailability, providing a rapid action and leading to a significant increase in cellular uptake rate [40]. Among the main factors able to affect nanoparticles particle size, by keeping the preparation conditions constant, formulation composition, i.e., type of lipids and surfactants used, may play an important role [41].

PDI is another important index of the physical stability of nanosuspensions, representing the width of particle size distribution. Low PDI values (<0.3) indicate a narrow size distribution, whereas PDI values greater than 0.5 are index of poorly homogeneous systems [42].

Finally, Zeta potential, i.e., the nanoparticles surface charge, is closely related to stability of the nanoparticles suspension, hindering aggregation phenomena [43,44]. In general, ZP values around ±30 mV are suggested for assuring good stability under storage of nanoparticles only electrostatically stabilized. However, ZP values around ±20 mV are considered to be enough for nanoparticles whose stability is the resultant of a combination of electrostatic and steric stabilization [45].

#### 3.3.1. Effect of Type and % of Surfactant on SLN Physicochemical Properties

**Figure 1:**
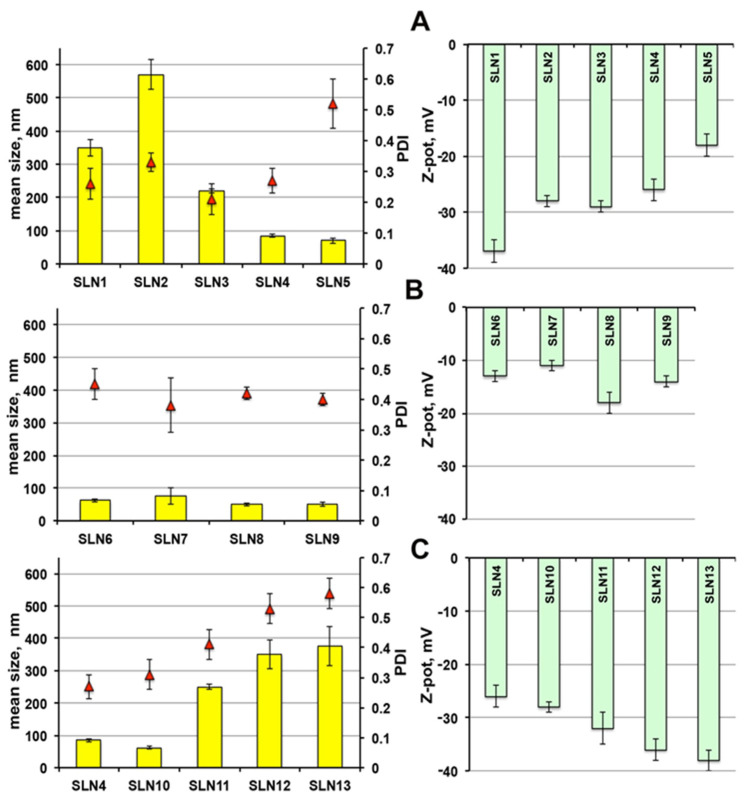
Physicochemical properties of empty SLNs in terms of mean size, polydispersity index (PDI) and Zeta potential: Scheme 1. SLN5 (**A**); SLN6-SLN9 (**B**); SLN10-SLN13 compared to SLN4 (**C**). See Table 2 for their composition.

As shown in Figure 1A, the mean diameter of SLNs obtained using Pluronic^®^ F68 (SLN_1_) was around 350 nm, significantly smaller than those with Tween^®^ 80 (SLN_2_) (above 550 nm). This finding, observed also by other authors [22,30,46], has been attributed to a different incorporation way of the two surfactant molecules in the external shell of the nanoparticles [47].

The replacement of such surfactants with an equal amount (1.5%) of Gelucire^®^ 44/14 (SLN_3_), gave rise to very homogeneous nanoparticles of smaller dimensions, around 220 nm. A rise up to 3.5% of Gelucire^®^ 44/14 content (SLN_4_) resulted in a further clear particle size decrease, up to around 85 nm, probably due to the higher surfactant/lipid ratio [48]; good PDI and satisfactory ZP values were maintained. A further reduction of the nanoparticles dimensions (around 70 nm) was observed by increasing Gelucire^®^ 44/14 content up to 7% (SLN_5_), but it was accompanied by a PDI increase and a ZP decrease. Therefore, only SLN_4_ formulation was selected for drug loading, together with SLN_1_ and SLN_2_ for comparison purpose.

The combined use of Gelucire^®^ 44/14 with Pluronic^®^ F68 or Tween^®^ 80 (SLN_6_-SLN_9_), resulted in all cases in nanoparticles of very reduced dimensions, ranging from 75 to 50 nm (Figure 1B). However, while an acceptable homogeneity was maintained, a sensible reduction of ZP was observed, index of potential physical instability of the colloidal dispersion. For this reason, this formulation series was discarded and not considered for further studies.

Finally, the series of SLN formulations containing a fixed 3.5% Gelucire^®^ 44/14 content and increasing amounts of the solid lipid Precirol^®^ ATO5 (Figure 1C) showed that, after an initial particle size reduction for SLN containing 7.5% of solid lipid (SLN_10_), a progressive increment of nanoparticles dimensions was instead observed when further rising its content, along with a PDI increase. This finding could be attributed to the concomitant decrease in the surfactant-to-lipid ratio [48]. Then, despite the favourable increment of the negative ZP value observed when increasing the Precirol^®^ ATO5 content, formulations SLN_11_, SLN_12_ and SLN_13_ were discarded and only SLN_10_ was selected for drug loading.

#### 3.3.2. Effect of Surfactant Type and Liquid Lipid Amount on NLC Physical-Chemical Properties

**Figure 2:**
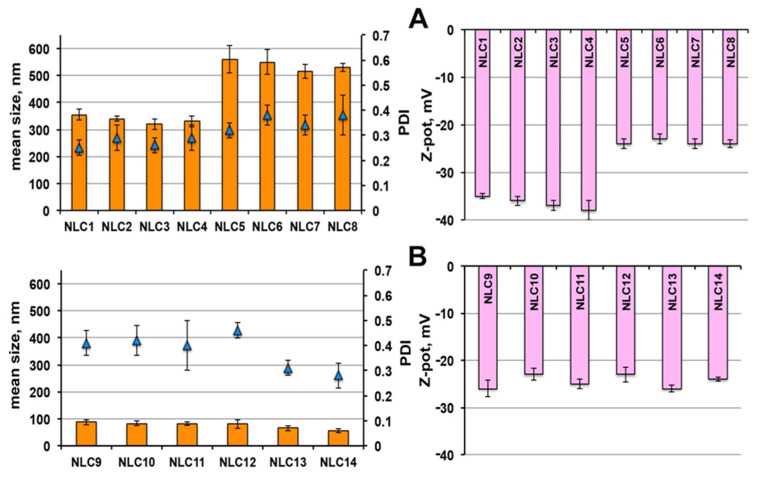
Physicochemical properties of empty NLCs in terms of mean size, polydispersity index (PDI) and Zeta potential: NLC_1_-NLC_8_ (**A**); NLC_9_-NLC_14_ (**B**). See Table 3 for their composition.

As shown in Figure 2A, even in the case of NLCs, the use as surfactant of Tween^®^ 80 (NLC_5_-NLC_8_) instead of Pluronic^®^ F68 (NLC_1_-NLC_4_), gave rise to nanoparticles of greater mean size (around 500 vs. 350 nm), in agreement with previous results [25]. Moreover, for each NLC series, no significant variations of their dimensions, homogeneity and ZP were found with increasing the liquid lipid amount up to 5%.

Interestingly, the use of 3.5% Gelucire^®^ 44/14 as surfactant (NLC_9_-NLC_12_) in place of Tween^®^ 80 or Pluronic^®^ F68 (Figure 2B), resulted in a marked reduction of nanoparticle dimensions (in all cases below 100 nm), with only a slight PDI increase. On the other hand, no advantages were observed in terms of nanoparticle size reduction or homogeneity improvement or ZP increase by enhancing the Transcutol^®^ HP content from 0.5% (NLC_9_) up to 5% (NLC_12_).

A further decrease in nanoparticles size, accompanied by an improvement in homogeneity, was instead found when using Gelucire^®^ 44/14 in combination with Pluronic^®^ F68 (NLC_13_) or Tween80 (NLC_14_), by keeping the liquid lipid amount constant at 0.5%. On the contrary, the kind of surfactant did not affect the ZP, which always ranged between −23 and −26 mV. These values can be considered sufficient to assure a good stability of the colloidal dispersions, in virtue of the presence of the steric-stabilizing surfactants [47].

Therefore, based on these results, and in order to minimize the use of Transcutol^®^ HP, formulations NLC_1_, NLC_5_ and NLC_9_ were selected for drug loading, containing, respectively, Pluronic^®^ F68, Tween^®^ 80 and Gelucire^®^ 44/14 as surfactant and all containing the lowest liquid lipid content (0.5%). Formulations NLC_13_ and NLC_14_, containing Gelucire^®^ 44/14 in mixture with Pluronic^®^ F68 and Tween^®^ 80, respectively, were also selected, due to their favourable properties in terms of very small particle size (around 60 nm), together with high homogeneity (PDI < 0.3) of the colloidal dispersion.

#### 3.3.3. Characterization of Drug-Loaded SLNs and NLCs

**Figure 3:**
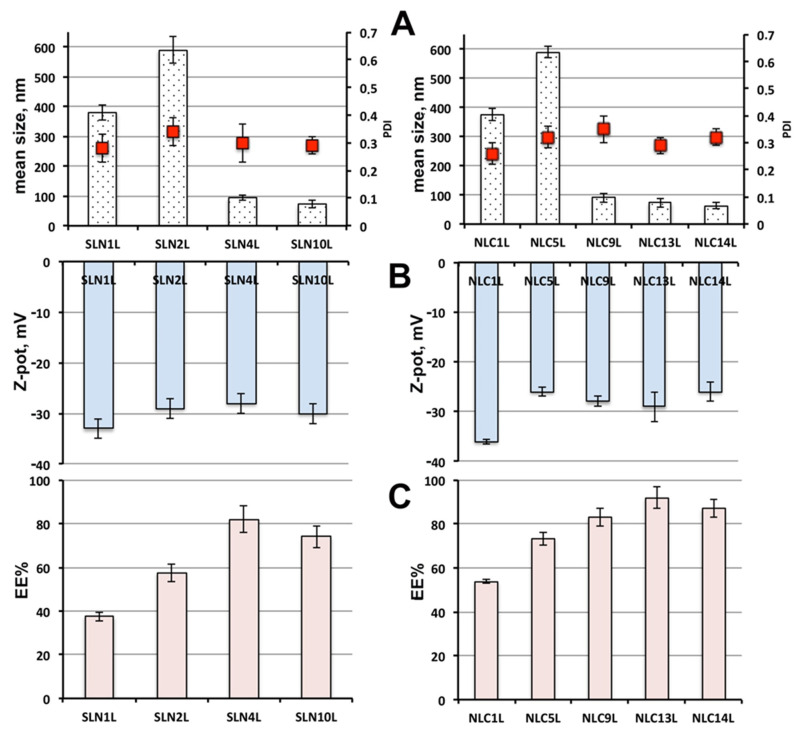
Physicochemical properties of the selected HCT-loaded SLN (left side, SLNnL) and NLC (right side, NLCnL) formulations: mean size and polydispersity index (PDI) (**A**), Zeta potential (**B**) and entrapment efficiency (EE%) (**C**).

The effect of drug loading on the physicochemical properties of the selected SLN and NLC formulations is presented in Figure 3A,B), where the letter L was added to the code of loaded formulations, to distinguish them from the corresponding empty ones. As expected, drug loading gave rise to a particle size increase, which was, however, limited, never exceeding 10% with respect to the corresponding empty nanoparticles. Moreover, no important variations were observed regarding the colloidal dispersion homogeneity, in terms of PDI values, or of the vesicle stability, in terms of surface charge, suggesting in all cases the obtainment of physically stable colloidal dispersion, substantially unaffected by drug loading.

As for the entrapment efficiency of SLN formulations (Figure 3C), the use as non-ionic surfactant of 3.5% Gelucire^®^ 44/14 (SLN_4_L), rather than 1.5% Pluronic^®^ F68 (SLN_1_L) or Tween^®^ 80 (SLN_2_L), resulted in an increase in EE% of 2.1 and 1.4 times, respectively, reaching about 80%. A smaller increase (1.8 and 1.2 times, respectively) was instead observed for SLN_10_L formulation, containing the same amount of Gelucire^®^ 44/14, but a higher content of the solid lipid Precirol^®^ ATO5 (7.5 vs. 5%), which probably negatively affected the drug incorporation ability of the nanoparticles.

Even in the case of NLC formulations, the replacement of Pluronic^®^ F68 (NLC_1_L) or Tween^®^ 80 (NLC_5_L) with 3.5% Gelucire^®^ 44/14 (NLC_9_L) enabled an entrapment efficiency improvement, by about 1.5 and 1.1 fold, respectively, overcoming 80%. A further EE% enhancement was obtained for NLC formulations containing Gelucire^®^ 44/14 in mixture with Pluronic^®^ F68 (NLC_13_L) or Tween^®^ 80 (NLC_14_L), which, respectively, approached and even exceeded 90%.

Finally, by comparing the results obtained with the different SLNs and NLCs formulations, rather similar findings were observed in terms of size, PDI and Zeta potential of the nanoparticles. On the contrary, the better performance of NLCs in terms of entrapment efficiency appeared evident, due to the less ordered structure of this kind of lipid nanoparticles, compared to SLNs, which enabled them to incorporate greater drug amounts.

### 3.4. Release Studies

**Figure 4:**
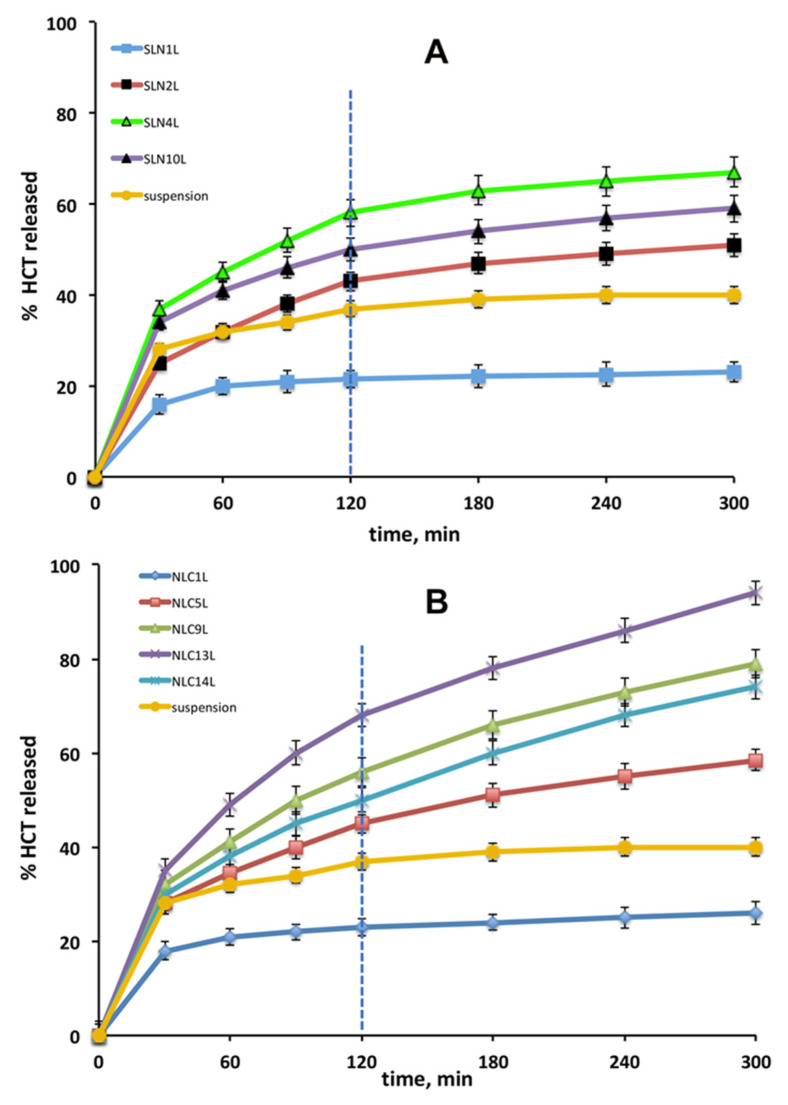
HCT release curves from the selected SLNnL (**A**) and NLCnL (**B**) formulations, at 37 °C for 2 h in pH 4.5 and 3 h in pH 6.8, together with that of the simple HCT suspension as reference.

The HCT release curves from the selected SLN and NLC formulations are shown in Figure 4, together with that from the simple drug suspension, for comparison purposes.

The HCT suspension exhibited an initial fast release phase, due to the diffusion of the fraction of already dissolved drug; however, it was rapidly followed by a slow release phase, due to the very low drug solubility, reaching about 40% drug released at the end of the test.

Regarding NLCs formulations (Figure 4B), similarly to what observed for SLNs formulations, NLCs containing Pluronic^®^ F68 (NLC_1_L) exhibited a reduced release rate compared to the drug suspension, while an opposite result was given by Tween^®^ 80-based NLC formulation (NLC_5_L), which reached about 60% drug released at 5 h. This behaviour was analogous to that previously reported for analogous NLCs formulations, containing castor oil instead of Transcutol^®^ HP as liquid lipid [25], and once again supported the critical role played by the surfactant type in affecting the drug release profile from lipid nanoparticles.

As found for SLNs formulations, the use of Gelucire^®^ 44/14 as surfactant (NLC_9_L) resulted in a clear improvement of the drug release rate, achieving nearly 80% of drug released at 5 h. Interestingly, a further release rate increase was given by NLC formulations containing Gelucire^®^ 44/14 in mixture with Tween^®^ 80 (NLC_13_L), which showed a total drug amount released at the end of the test of about 95%. On the contrary, a slight decrease in drug delivery rate was observed when Pluronic^®^ F68 was added in mixture to Gelucire^®^ 44/14 (NLC_14_L).

Finally, by comparing the global results obtained with the two series of lipid nanoparticles formulations, it appears evident that more regular and faster release profiles were generally provided by NLC than from SLN formulations. In particular, the best NLC formulations allowed a drug release rate 1.4 times higher than the best SLN formulation. This effect may be ascribed to the different physical state of the lipids in the two kinds of nanoparticles. In fact, in the case of SLNs, the lipids are more densely and ordinately packed, thus hindering the drug release, while the less ordered and less crystalline structure of NLC can facilitate and improve drug delivery [49].

Kinetic evaluation of drug release profiles from the different SLNs and NLCs was also performed, in order to obtain some insight about the prevalent mechanisms governing drug release from the delivery system. Release data were fitted into the most common release kinetic models: zero order, first order, Higuchi and Korsmeyer–Peppas models. The obtained values of correlation coefficients (R^2^) and release exponent (*n*), summarized in Table 4, indicated that SLNs followed Korsmeyer–Peppas release kinetic, while NLCs release data well fitted with both Higuchi and Korsmeyer–Peppas kinetic models. In any case, *n* values <0.5 were obtained in all cases, indicating a predominantly diffusion-controlled mechanism of drug release. Moreover, the initial burst release observed during the first 30 min can be due to the diffusion of drug present on the nanoparticle outer surface, and might provide a fast onset of action.

Considering all of the obtained results, in terms of physico-chemical properties, entrapment efficiency and drug release behaviour, SLN_4_L, NLC_9_L, NLC_13_L, and NLC_14_L were ultimately selected as the best formulations of the two series of colloidal dispersions and subjected to further studies to evaluate their stability under storage and their actual safety.

### 3.5. Storage Stability Studies

The physical stability of the selected SLN and NLC formulations was monitored during 6 months of storage at 4 °C. The simple visual inspection did not evidence any phenomenon of drug precipitation, or crystallization process or formation of mold.

**Figure 5:**
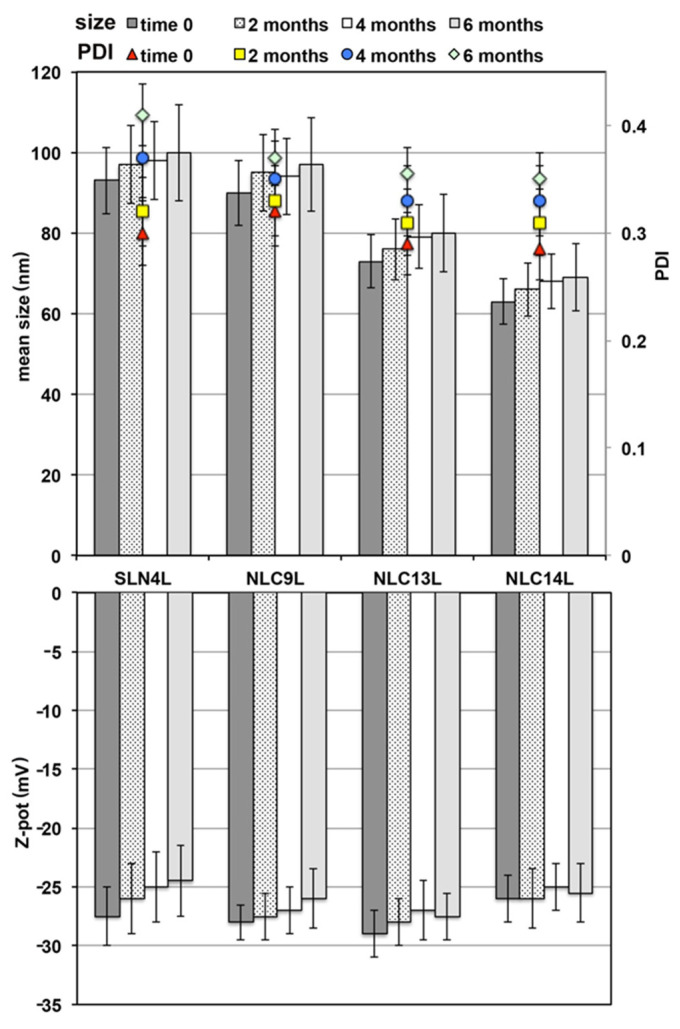
Effect of storage at 4 ± 1 °C on mean size, PDI and Zeta potential of the selected SLN and NLC formulations.

The physicochemical properties of the colloidal dispersions were evaluated in terms of variations in mean dimensions, homogeneity (PDI) and surface charge of the nanoparticles (Figure 5). In all cases, no significant changes in the values of such parameters (*p* > 0.05) were detected during the whole storage period with respect to the freshly prepared systems. Thus, these results were considered indicative of the good physical stability of all the colloidal dispersion systems considered.

At the end of the storage period, a check of the EE% of the selected formulations was also performed. In the case of all NLC formulations, only a slight, not significant (*p* > 0.05) reduction was observed, that never exceeded 5%. On the contrary, a more evident and significant (*p* < 0.05) decrease in the amount of encapsulated drug was found for SLN_4_L formulation, which reached an about 15% of drug leakage after 6 months storage at 4 °C. These results confirmed the lower tendency to drug expulsion phenomena during the storage of NLCs with respect to SLNs, in virtue of their less ordered nature, due to the presence of the liquid lipid within the solid lipid structure [24].

### 3.6. Cytotoxicity and Cellular Uptake Studies

**Figure 6:**
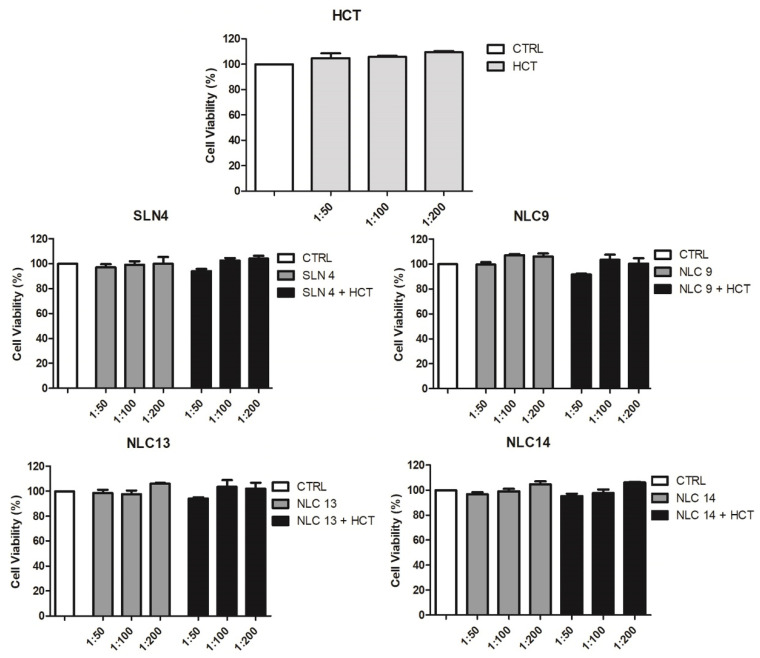
Cytotoxicity on CaCo-2 cell line expressed as cell viability % of pure HCT, empty and drug-loaded SLN4, empty and drug-loaded NLC9, NLC13, NLC14 at different dilutions (1:50, 1:100, 1:200) after 24 h exposure.

The cytotoxicity of the developed SLN and NLC formulations was assessed by MTS cell assay. Cell viability was determined after 24 h exposure of Caco-2 cells to empty or HCT-loaded SLN and NLC formulations or to a solution of plain drug, all at three different dilutions. The results of this study, reported in Figure 6 as a percentage of cell viability compared to that of control cells exposed to the solvent alone, proved that none of the evaluated SLN and NLC formulations, both empty or drug-loaded, exhibited a cytotoxicity, at all dilutions tested. In fact, in all cases, a cell viability close to 100% was maintained, similar to that of the control (pure solvent) or of the HCT simple solution. A slight reduction of up to approximately 95% was observed only for the drug-loaded NLC_9_ and NLC_13_ formulations, only at the lowest dilution (1:50) but this effect was not statistically significant compared to the control (*p* > 0.05). These results further highlighted the importance of a proper surfactants selection. Biocompatible surfactants are in fact highly preferable to minimize any potential surfactant-induced toxicity of lipid nanocarriers [50].

**Figure 7:**
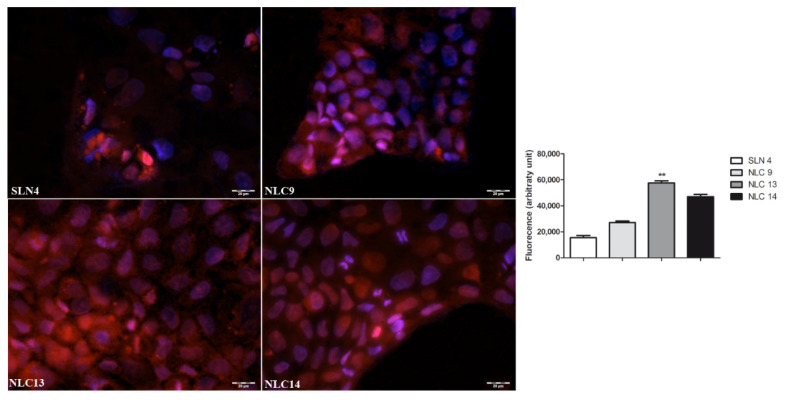
Cellular uptake of rhodamine-labeled SLN and NLC in Caco-2 cells after 1 h of exposure at 37 °C. Nuclei stained with DAPI. Final magnification 20×, scale bar 50 µm. Results expressed as mean ± SEM, *n* = 10; ** *p* < 0.01 vs. SLN4 (Kruskal–Wallis test and Dunn’s multiple comparisons test).

Cellular uptake experiments were performed to test the actual internalization of SLN and NLC formulations into intestinal epithelium. To this aim, Caco-2 cells were incubated with SLN and NLCs labeled with rhodamine as fluorescent dye. After 1 h of exposure, an evident intracellular staining was observed in all cases, demonstrating that all formulations were able to enter Caco-2 intestinal cells, even though some differences in intensity of the effect were observed among the different formulations, NLC13 and NLC14 being the most effective ones, probably due to their smaller size and/or the combination of two different surfactants (Figure 7).

## 4. Conclusions

Careful investigations about the choice of the most effective solid and liquid lipid components as well as surfactants for the development of new SLN and NLC-based HCT pediatric formulations were successful in further improving their performance with respect to previously developed formulations [22,25] in terms of particle size, entrapment efficiency, drug release properties and storage stability.

In fact, in the case of SLN formulations, the use of Gelucire^®^ 44/14 (rather than Pluronic^®^ F68 or Tween^®^ 80) yielded a marked reduction of the particle size up to 95–75 nm (with respect to around 600–400 nm), and at the same time an improvement in entrapment efficiency, reaching about 80% (with respect to 40 or 60%, respectively) as well as in drug release rate, reaching about 65% of HCT released after 300 min (versus 20 or 50%, respectively). On the other hand, in case of NLC formulations, the use of Gelucire^®^ 44/14, alone (NLC_9_L), or in combination with Pluronic^®^ F68 or Tween^®^ 80 (NLC_13_L and NLC_14_L, respectively), allowed also in this case a dramatic reduction of particle size up to 90, 75 and 65 nm, respectively (compared to around 400 or 600 nm of NLC_1_L or NLC_5_L containing PluronicF68 or Tween^®^ 80 alone, respectively), maintaining a similar entrapment efficiency, around 90%, while improving drug release rate, reaching more than 90% of HCT released after 300 min (versus 25 or 60%).

A comparison between the selected SLN (SLN_4_L and SLN_10_L) and NLC (NLC_9_L, NLC_13_L and NLC_14_L) formulations confirmed the superior performance of the latter in terms of drug entrapment efficiency and drug release behaviour. Similar results were instead obtained in stability studies, where all the examined formulations maintained almost unchanged their properties in terms of particle size, PDI and zeta potential, without any apparent variations of organoleptic properties nor mold formation after 6 months storage at 4 °C. However, a more evident drug leakage phenomenon was found for SLN (15%) than for all NLCs (<5%)

Importantly, none of the new developed SLN and NLC HCT formulations showed signs of cytotoxicity on Caco-2 cells, used as model of human intestinal cells, confirming the safety of the employed excipients. Finally, cellular uptake studies proved that all the selected formulations were able to enter Caco-2 intestinal cells thus increasing HCT intestinal permeability.

## Figures and Tables

**Table 1:** HCT solubility in melted solid lipids (65 °C), and in liquid lipids heated at 65 °C in terms of solution transparency or turbidity (100 or 200 mg drug added to 5 g of melted solid lipid or 5 mL of liquid lipid).

Solid Lipid	Appearance of the Solution after Addition of		Liquid Lipid	Appearance of the Solution after Addition of	
	100 mg HCT	200 mg HCT		100 mg HCT	200 mg HCT
Precirol^®^ ATO5	transparent	transparent	Labrafac^®^ WL1349	turbid	
Compritol^®^ 888ATO	turbid		Labrasol^®^	transparent	turbid
Geleol^®^	transparent	turbid	Labrasol^®^ ALF	transparent	turbid
Cetyl Palmitate	turbid		Transcutol^®^ HP	transparent	transparent
Tripalmitin	turbid		Transcutol^®^ P	transparent	transparent
Imwitor^®^ 491	transparent	turbid	Miglyol^®^ 810N	turbid	
			Miglyol^®^ 812	turbid	

**Table 2:** Composition (% *w*/*w*) of the different SLN colloidal dispersions in water.

Code	Solid Lipid	Surfactant Type		
	Precirol ATO5	Pluronic F68	Tween 80	Gelucire
SLN_1_	5	1.5	-	-
SLN_2_	5	-	1.5	-
SLN_3_	5	-	-	1.5
SLN_4_	5	-	-	3.5
SLN_5_	5	-	-	7.0
SLN_6_	5	1.5	-	5
SLN_7_	5	-	1.5	5
SLN_8_	5	1.5	-	6
SLN_9_	5	-	1.5	6
SLN_10_	7.5			3.5
SLN_11_	10			3.5
SLN_12_	15			3.5
SLN_13_	20			3.5

**Table 3:** Composition (% *w/w*) of the different NLC colloidal dispersions in water.

Code	Solid Lipid	Liquid Lipid	Surfactant Type		
	Precirol ATO5	Transcutol HP	Pluronic F68	Tween 80	Gelucire
NLC_1_	5	0.5	1.5	-	-
NLC_2_	5	1	1.5	-	-
NLC_3_	5	3	1.5	-	-
NLC_4_	5	5	1.5	-	-
NLC_5_	5	0.5	-	1.5	-
NLC_6_	5	1	-	1.5	-
NLC_7_	5	3	-	1.5	-
NLC_8_	5	5	-	1.5	-
NLC_9_	5	0.5	-	-	3.5
NLC_10_	5	1	-	-	3.5
NLC_11_	5	3	-	-	3.5
NLC_12_	5	5	-	-	3.5
NLC_13_	5	0.5	1.5	-	3.5
NLC_14_	5	0.5	-	1.5	3.5

**Table 4:** Correlation coefficients (R^2^) and release exponent (*n*) values obtained from the different kinetic models for SLNs (upper) and NLCs (down) formulations.

Kinetic Model	SLN_1_L	SLN_2_L	SLN_4_L	SLN_10_L	
Zero order	0.9239	0.8713	0.8578	0.9236	
First order	0.927	0.8989	0.8995	0.9483	
Higuchi	0.964	0.9313	0.9214	0.9690	
Kormeyer-Peppas	0.9842	0.9538	0.9509	0.9862	
Kormeyer-Peppas (*n*)	0.08	0.28	0.24	0.22	
**Kinetic model**	**NLC_1_L**	**NLC_5_L**	**NLC_9_L**	**NLC_13_L**	**NLC_14_L**
Zero order	0.9771	0.9527	0.9675	0.9639	0.9853
First order	0.9797	0.9756	0.9881	0.9751	0.9897
Higuchi	0.9955	0.9866	0.9940	0.9913	0.9992
Kormeyer-Peppas	0.9958	0.9939	0.9963	0.9933	0.9994
Kormeyer-Peppas (*n*)	0.13	0.33	0.40	0.39	0.42

## Data Availability

Not applicable.
